# Development and validation of a nomogram for predicting sever cancer-related fatigue in patients with cervical cancer

**DOI:** 10.1186/s12885-024-12258-x

**Published:** 2024-04-18

**Authors:** ZhiHui Gu, ChenXin Yang, Ke Zhang, Hui Wu

**Affiliations:** Department of Social Medicine, School of Health Management, China Medical University, No.77 PuHe Road, Shenyang North New District, 110122 Shenyang, Liaoning People’s Republic of China

**Keywords:** Nomogram, Cancer-related fatigue, Coping styles, Perceived Social Support, Sense of coherence

## Abstract

**Objective:**

Cancer-related fatigue (CRF) has been considered the biggest influencing factor for cancer patients after surgery. This study aimed to develop and validate a nomogram for severe cancer-related fatigue (CRF) patients with cervical cancer (CC).

**Methods:**

A cross-sectional study was conducted to develop and validate a nomogram (building set = 196; validation set = 88) in the Department of Obstetrics and Gynecology of a Class III hospital in Shenyang, Liaoning Province. We adopted the questionnaire method, including the Cancer Fatigue Scale (CFS), Medical Uncertainty in Illness Scale (MUIS), Medical Coping Modes Questionnaire (MCMQ), Multidimensional Scale of Perceived Social Support (MSPSS), and Sense of Coherence-13 (SOC-13). Binary logistic regression was used to test the risk factors of CRF. The R4.1.2 software was used to develop and validate the nomogram, including Bootstrap resampling method, the ability of Area Under Curve (AUC), Concordance Index (C-Index), Hosmer Lemeshow goodness of fit test, Receiver Operating Characteristic (ROC) curve, Calibration calibration curve, and Decision Curve Analysis curve (DCA).

**Results:**

The regression equation was Logit(*P*) = 1.276–0.947 Monthly income + 0.989 Long-term passive smoking − 0.952 Physical exercise + 1.512 Diagnosis type + 1.040 Coping style − 0.726 Perceived Social Support − 2.350 Sense of Coherence. The C-Index of the nomogram was 0.921 (95% CI: 0.877$\sim$0.958). The ROC curve showed the sensitivity of the nomogram was 0.821, the specificity was 0.900, and the accuracy was 0.857. AUC was 0.916 (95% CI: 0.876$\sim$0.957). The calibration showed that the predicted probability of the nomogram fitted well with the actual probability. The DCA curve showed when the prediction probability was greater than about 10%, the benefit of the nomogram was positive. The results in the validation group were similar.

**Conclusion:**

This nomogram had good identifiability, accuracy and clinical practicality, and could be used as a prediction and evaluation tool for severe cases of clinical patients with CC.

## Background

Cervical cancer (CC) has become one of the most susceptible and lethal tumors for women due to the increase of sexually transmitted diseases. GLOBAOCAN report showed that in 2020, there would be 600,000 new cases of CC worldwide, and the number of deaths due to CC would reach 340,000 [1]. CC ranked the fourth in the number of new cases of women in the world [2]. CC has become an important public health problem. At present, surgery and chemotherapy are the most commonly used treatment methods for CC, which can improve the overall survival rate and prolong the life expectancy of patients, but it is difficult to avoid the harm caused by surgical trauma, complications and side effects of radiotherapy and chemotherapy.

Cancer-related fatigue (CRF) often runs through all stages of radiotherapy, chemotherapy and even hospice care for cancer patients [3]. Piper first proposed the concept of CRF in 1987, defining it as a subjective, specific and systematic feeling of excessive fatigue, which was closely related to the cancer itself and its therapeutic factors [4]. Ma showed that the overall incidence rate of CRF among 144,813 cancer patients was 52%, and the number of patients with moderate fatigue was significantly higher than that of mild and severe fatigue [5]. Cancer survivors reported that CRF was a serious and destructive symptom that can last for months to years after treatment [6]. Gernier et al. followed up 45 patients with CC and found that the proportion of physical fatigue and mental fatigue was 45.2% and 37.8% respectively [7]. Al Maqbali et al. found that the incidence rate of CRF during treatment and within three months after treatment was as high as 62.0% and 50.1%, respectively, and 43% of the survivors still had fatigue symptoms of varying degrees [8]. This demonstrated that CRF could occur at various stages of cancer treatment. Research has shown that 60–90% of cancer patients who received treatment experience symptoms of CRF, including physical weakness, silence, and functional impairment [9]. Compared with patients without CRF, patients with CRF had relatively poorer quality of life, more prominent symptoms of depression and anxiety, and severe physical and cognitive dysfunction [10]. Overall, severe CRF could affect daily activities [11], and lead to depressive symptoms [12], poor quality of life, lack of vitality, work difficulties, relationship issues, emotional distress [13], and even affect therapeutic compliance and clinical outcomes including recurrence and mortality [14].

Clinical prediction models (CPMs) are used to evaluate the probability of a specific subject suffering from a certain disease or having a certain clinical result in the future [15]. CPMs calculate the probability of a certain disease or complication in the current state according to the patient’s clinical symptoms, disease characteristics and other relevant data information [16]. The prediction model of CRF constructed by Meglio et al. found that age, BMI, current smoking behavior, anxiety, insomnia, and pain during diagnosis were predictive factors, and the accuracy of the model was very high [17]. Lee et al. also constructed a random forest regression model for CRF in patients with breast cancer, and found a subset of genes with more predictive significance, such as peroxygenase-5, connector protein, and the accuracy of the model was high [18]. Huang et al. constructed a back-propagation artificial neural network model to predict the risk of moderate to severe CRF in colorectal cancer patients and found surgery, complications, hypokalaemia, albumin, neutrophil percentage, pain, sleep quality, anxiety, depression and nutrition were predictive factors [19].

As a type of CPMs, a nomogram have been widely used as a prediction method in oncology in recent years [20–23]. It meets the requirements of integrated models, plays a role in promoting personalized healthcare, and is convenient for clinical doctors to use for prognosis prediction [24]. A nomogram refers to a quantitative analysis chart that represents the functional relationship between multiple variables using a set of disjoint line segments in planar coordinates [24]. Its advantage is that it can directly use the graph to calculate the value of a certain variable, such as the patient’s indicator score or survival probability [24]. The most common one is the probability nomogram, which determines the probability of a specific event occurring in an individual, such as disease occurrence, recurrence, and prognosis (such as death) [25]. Essentially, a nomogram is a visualization of the results of a regression equation, commonly used for displaying the results of logistic regression or COX regression [26]. Based on the regression results, multiple line segments are drawn in specific proportions, and through plotting, the disease risk or survival probability of an individual can be conveniently calculated [26]. Many studies have used a nomogram to predict the probability of fatigue occurrence in different populations, and have validated the accuracy of the nomogram [27–30].

However, a nomogram of sever CRF in patients with CC was rarely reported. Therefore, we included the factors that have been confirmed by previous studies that might affect CRF, including age, economic status, exercise status, clinical status and psychological variables [27–32]. This study aimed to develop and validate a scientific, accurate and convenient new assessment tool for the prediction of severe CRF in patients with CC, so as to help clinical workers identify high-risk groups with severe CRF in CC as early as possible.

## Materials and methods

### Study design

We conducted a cross-sectional study and adopted a face-to-face questionnaire survey in the Department of Obstetrics and Gynecology of a Class III hospital in Shenyang, Liaoning Province from May 2021 to March 2022. Our study was conducted in accordance with the Transparent Reporting of a Multivariable Prediction Model for (TRIPOD) checklist [33]. Medical staff used the nomogram constructed in this study to assess severe CRF in patients with CC admitted for treatment, including questionnaire surveys or inquiry methods.

### Sample size calculation

The development of a nomogram requires selecting a group of influencing factors of outcome variables as predictors, and then selecting appropriate models to screen for statistically significant and important clinical variables based on the data type of outcome variables, thereby forming a nomogram and evaluating it. According to logistic regression analysis, the estimated sample size is at least 5$\sim$10 times the number of variables. This study included a total of 21 evaluation factors, with a sample size formula of 21*(5$\sim$10) = 105$\sim$210 cases [34]. Considering the allocation principle of 70% and 30% participants in the model development group and model validation group, it was reasonable to calculate the total sample size (105$\sim$210)/0.7 = 150$\sim$300. This meant that the sample size of the model development group should be at least 150, and the sample size of the validation group should be at least 65. This study ultimately collected data from 284 patients with CC. According to the allocation principle of 70% and 30%, the sample size of the model development group was 196 cases, and the sample size of the validation group was 88 cases.The first 70% of the case data (*N* = 196) was included in the model development group and the last 30% of the case data (*N* = 88) was included in the validation group according to the order of inclusion in the study. The patient data of the model development group was used for the development and internal evaluation of the risk assessment model and used to establish the prediction probability for the patients in the validation group.

The inclusion criteria were: (1) patients with primary CC confirmed by pathology; (2) aged ≥ 18 years old; (3) communicate and fill in questionnaires independently; (4) know the illness of themselves; (5) volunteer to participate in the investigation and sign the informed consent. Exclusion criteria for study subjects: (1) patients with other malignant tumors at the same time; (2) patients with a history of psychiatric diseases or mental retardation; (3) patients who had received psychotherapy or intervention within one year.

### Measurement of CRF

The Cancer Fatigue Scale (CFS) was designed by Okuyama and validated by 307 cancer patients [35]. In this study, we used the Chinese version of the CFS scale translated by Fengling Zhang [36]. It includes 15 items, with a total score of ≤ 5 for no fatigue, 6–15 for mild fatigue, 16–30 for moderate fatigue, and 31–60 for severe fatigue. The scale has been used in different cancer patients. It has been verified that the scale was simple and easy to complete, even for patients with advanced cancer.

### Risk factors for severe CRF

We included 20 risk factors for severe CRF. There were 16 demographic and clinical factors, including age, body mass index, marital status, education level, occupation, monthly income, long-term passive smoking, physical exercise, dietary characteristics, menopause, diagnosis type, tumor stage, treatment, and so on. There were 4 psychological factors, including uncertainty of illness, coping styles, perceived social support and sense of coherence. The meaning of the variables was detailed in Table 1.

**Table 1:** Variable declaration

Factors	Variables	Meaning
severe CRF	Y	Score: ≤30 = 0, 31–60 = 1 (severe CRF)
Age	X_1_	Years: ≤45 = 0, 46–55 = 1, > 55 = 2
Marital status	X_2_	Unmarried = 0, Married = 1
Education level	X_3_	Primary school and below = 0, Junior high school = 1, High school = 2, College/university or above = 3
Occupation	X_4_	Retired personnel = 0, State and public institutions = 1, Staff and workers of enterprise = 2, Self-employed personnel = 3, Unemployed person = 4
Per capita monthly income	X_5_	CNY: ≤2000 = 0, 2001–4000 = 1, 4001–5000 = 2, > 5000 = 3
Place of residence	X_6_	City = 0, Countryside = 1
Long-term passive smoking	X_7_	Expose to the smoke environment caused by smokers at least 4 times a week, for more than 15 min per day, and persist for a long time: No = 0, Yes = 1
BMI	X_8_	17$\sim$24 = 1, > 24 = 2
Physical exercise	X_9_	At least 30 min each time: Never = 0, Once/week = 1, ≥ 2–3 times/week = 2
Breakfast	X_10_	Never = 0, Occasionally = 1, Often = 2
Coffee consumption	X_11_	Never/Occasionally = 0, Often/Daily = 1
Menopause	X_12_	No = 0, Yes = 1
Diagnostic type	X_13_	New diagnosis = 0, Recrudescence = 1
Lymph node metastasis	X_14_	No = 0, Yes = 1
Cancer stage	X_15_	I=0, II=1, III+IV=2
HPV infection	X_16_	No = 0, Yes = 1, Not checked = 2
Uncertainty of Illness	X_17_	Score: 25$\sim$58 = 0 (Low), 59$\sim$125 = 1 (Medium/High)
Coping Styles	X_18_	Propensity to score: Face = 0, Avoid = 1, Yield = 2
Perceived Social Support	X_19_	Score: 12$\sim$36 = 0 (Low), 37$\sim$60 (Medium) = 1, 61$\sim$84 (High) = 2
Sense of Coherence	X_20_	Score: 13$\sim$63 (Low) = 0, 64$\sim$91 (Medium/High) = 1

## Measurement of risk factors

### Demographic and clinical characteristics

The self-made general situation questionnaire was used.

### Uncertainty in illness

The Medical Uncertainty in Illness Scale (MUIS) was developed by Michel and Braden under the guidance of the theory of medical uncertainty to assess the uncertainty level of adult patients in five aspects: symptoms, diagnosis, relationship with caregivers, treatment and prognosis [37]. In this study, we used the Chinese version of the MUIS scale translated by Zengjie Ye [38]. It has 25 items in total and adopts the Likert five level scoring method. The scale has a score range of 25–125 points, which can be divided into three levels, namely, low level 25–58 points, medium level 59–91 points and high level 92–125 points.

### Coping modes

The Medical Coping Modes Questionnaire (MCMQ) was developed by Feifel and was applicable to patients with various diseases [39]. In this study, we used the Chinese version of the MCMQ scale translated by Xiaohong Shen and Qianjin Jiang [40]. It contains three dimensions: facing (8 items), avoiding (7 items) and yielding (5 items). There are 20 items in total, and the 4-level scoring method was used. The total score range was 20–80. The higher the score, the more inclined the individual was to adopt this coping style.

### Perceived social support

The Multidimensional Scale of Perceived Social Support (MSPSS), developed by Zimet [41]. In this study, we used the Chinese version of the MSPSS scale translated by Qianjin Jiang [42]. It includes 12 items and 3 dimensions (friend support, family support, and important others support). The score of each item ranges from 1 to 7 points. The total score of the scale ranges from 12 to 84 points, which is divided into three levels. 12 to 36 points is low support, 37 to 60 points is intermediate support, and 61 to 84 points is high support.

### Sense of coherence

The Sense of Coherence-13 (SOC-13) was a simplified version of SOC-29 by Antonovsky [43]. In this study, we used the Chinese version of the SOC-13 scale translated by Shiu [44]. It includes three dimensions: comprehensibility, controllability and sense of meaning SOC-13 uses a 7-level scoring method, with a total score ranges of 13$\sim$91 points, of which 13–63 points is low, 64–79 points is medium, and 80–91 points is high. Compared with SOC-29, the simplified version of SOC-13 is more widely used.

### Statistical methods

IBM SPSS Statistics 26 was used for statistics and analysis, which included chi-square test and Binary logistic regression. The method of deleting cases to handle missing data. The R4.1.2 software was used to develop and verify the nomogram. The R packages used in this study included “Rms 6.3.0 (Nomograms, Calibration curve)”, “DescTools 0.99.46 (C-Index)”, “ROCit 2.1.1” (ROC analysis), “ResourceSelection 0.3.5” (Hosmer-Lemeshow test), “Rmda 1.6” (DCA analysis). We used bootstrap resampling method, the ability of AUC and C-Index evaluation models to distinguish patients with severe CRF from patients with mild CRF [45]. The accuracy of the model was evaluated with Hosmer Lemeshow goodness of fit test and Calibration calibration curve, and the clinical practicability of the model was evaluated with DCA curve analysis results, so as to complete the internal evaluation of the model [45]. Finally we used the established prediction model for severe CRF of CC patients to establish the prediction probability for each patient in the validation group. Combined with the actual situation of the patients in the validation group who had severe CRF, the ROC curve, calibration curve and DCA curve were drawn to complete the validation of the nomogram. We adopted double-sided statistical test, the testing level was taken as α = 0.05.

## Results

### Single factor analysis of severe CRF

Severe CRF was used as the dependent variable. Table 2 showed the results of single factor analysis. Per capita monthly income (*P* < 0.001), long-term passive smoking (*P* < 0.001), physical exercise (*P* < 0.001), diagnosis type (*P* = 0.001), uncertainty in illness (*P* = 0.018), coping style (*P* < 0.001), perceived social support (*P* < 0.001), and sense of coherence (*P* < 0.001) were the influencing factors of severe CRF.

**Table 2:** The distribution of demographic, clinical, and psychological characteristics among CRF

Variables	N(%)	Severe CRF		χ^2^	p
		No	Yes		
*Demographic*					
X1 Age (Years)				0.501	0.778
≤ 45	59(30.1)	31	28		
46–55	47(24.0)	25	22		
> 55	90(45.9)	43	47		
X2 Marital status				1.696	0.193
Unmarried	15(7.7)	10	5		
Married	181(92.3)	89	92		
X3 Education level				6.441	0.092
Primary school and below	12(6.1)	9	3		
Junior high school	116(59.2)	51	65		
High school	47(24.0)	28	19		
College/university or above	21(10.7)	11	10		
X4 Occupation				2.104	0.717
Retired personnel	122(62.3)	65	57		
State and public institutions	10(5.1)	6	4		
Staff and workers of enterprise	24(12.2)	10	14		
Self-employed personnel	10(5.1)	4	6		
Unemployed person	30(15.3)	14	16		
X5 Per capita monthly income				35.480	< 0.001
≤ 2000 CNY	37(18.9)	13	24		
2001–4000 CNY	89(45.5)	51	38		
4001–5000 CNY	33(16.8)	5	28		
> 5000 CNY	37(18.8)	30	7		
X6 Place of residence				1.605	0.205
City	151(77.0)	80	71		
Countryside	45(23.0)	19	26		
X7 Long-term passive smoking				16.294	< 0.001
No	115(58.7)	72	43		
Yes	81(41.3)	27	54		
X8 BMI				0.881	0.348
17$\sim$24	153(78.1)	80	73		
> 24	43(21.9)	19	24		
X9 Physical exercise				32.294	< 0.001
Never	126(64.3)	47	79		
Once/week	32(16.3)	18	14		
≥ 2–3 times/week	38(19.4)	34	4		
X10 Breakfast				2.316	0.314
Never	17(8.7)	7	10		
Occasionally	20(10.2)	13	7		
Often	159(81.1)	79	80		
X11 Coffee consumption				1.032	0.310
Never/Occasionally	171(87.2)	84	87		
Often/Daily	25(12.8)	15	10		
*Disease*					
X12 Menopause				1.418	0.234
No	54(28.0)	31	23		
Yes	142(72.0)	68	74		
X13 Diagnostic type				10.466	0.001
New diagnosis	166(84.7)	92	74		
Recrudescence	30(15.3)	7	23		
X14 Lymph node metastasis				1.344	0.246
No	138(70.4)	66	72		
Yes	58(29.6)	33	25		
X15 Cancer stage				5.595	0.061
I	43(21.9)	21	22		
II	113(57.7)	64	49		
II+III	40(20.4)	14	26		
X16 HPV infection				5.524	0.063
No	127(64.8)	72	55		
Yes	43(21.9)	17	26		
Not checked	26(13.3)	10	16		
*Psychology*					
X17 Uncertainty of Illness				5.582	0.018
Low	42(21.4)	28	14		
Middle/High	154(78.6)	71	83		
X18 Coping Styles				44.637	< 0.001
Face	86(43.8)	66	20		
Avoid	57(29.1)	13	44		
Yield	53(27.1)	20	33		
X19 Perceived Social Support				33.628	< 0.001
Low	32(16.3)	10	22		
Middle	97(49.5)	36	61		
High	67(34.2)	53	14		
X20 Sense of Coherence				47.714	< 0.001
Low	132(67.3)	44	88		
Middle/High	64(32.7)	55	9		

### Multivariate logistic regression analysis of severe CRF

Table 3 showed the results of logistic regression analysis. Long-term passive smoking (*β* = 0.989, OR = 2.688, *P* = 0.023), tumor recurrence (*β* = 1.512, OR = 4.534, *P* = 0.012), and coping styles of yield (*β* = 1.040, OR = 2.829, *P* < 0.001) were independent risk factors for severe CRF. Per monthly income > 5000CNY (*β*=-0.947, OR = 0.388, *P* = < 0.001), physical exercise ≥ 2–3 times/week (*β*=-0.952, OR = 0.386, *P* = 0.001), higher perceived social support (*β*=-0.726, OR = 0.484, *P* = 0.030), and higher sense of coherence (*β*=-2.350, OR = 0.095, *P* < 0.001) were protective factors for severe CRF.

**Table 3:** Multivariate analysis of severe CRF

Influence factor	β	S.E.	Waldχ2	P	OR	95%CI
Per capita monthly income	−0.947	0.241	4.230	< 0.001	0.388	0.242$\sim$0.622
Long-term passive smoking	0.989	0.436	6.859	0.023	2.688	1.143$\sim$6.323
Physical exercise	−0.952	0.295	8.594	0.001	0.386	0.216$\sim$0.689
Diagnostic Type	1.512	0.603	4.591	0.012	4.534	1.390$\sim$14.788
Uncertainty of Illness	−0.165	0.623	0.070	0.791	0.848	0.250$\sim$2.873
Coping Style	1.040	0.259	14.963	< 0.001	2.829	1.703$\sim$4.699
Perceived Social Support	−0.726	0.335	5.966	0.030	0.484	0.251$\sim$0.932
Sense of Coherence	−2.350	0.540	23.257	< 0.001	0.095	0.033$\sim$0.275
Constant	1.276			0.043		

*Note*: Per capita monthly income:>5000 CNY vs. ≤ 2000 CNY; Physical exercise: ≥ 2–3 times/week vs. Never; Diagnosis type: recurrence vs. new diagnosis; Coping Style: yield vs. face; Perceived Social Support: high vs. low; Sense of Coherence: medium/high vs. low

### Establishment and internal evaluation of the nomogram for severe CRF

**Fig. 1:**
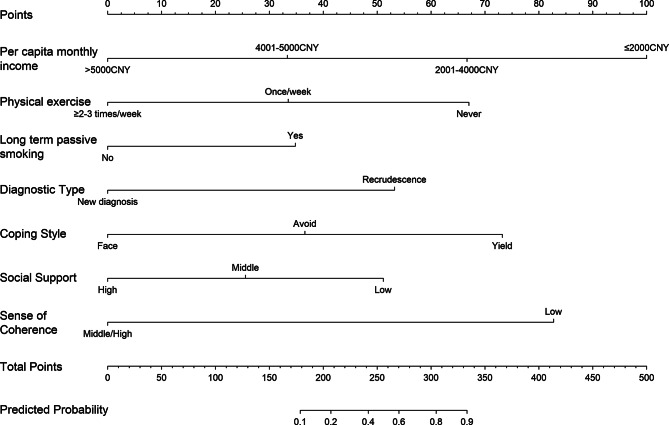
Nomogram for severe CRF

According to the logistic regression coefficient in Table 3, the regression equation of the nomogram for severe CRF in patients with CC can be obtained as follows: Logit(*P*) = 1.276–0.947 Monthly income + 0.989 Long-term passive smoking − 0.952 Physical exercise + 1.512 Diagnosis type + 1.040 Coping style − 0.726 Perceived Social Support − 2.350 Sense of Coherence. The model was visualized in the form of this nomogram, as shown in Fig. 1. According to the nomogram, the corresponding score values for each prediction indicator were obtained, added up the corresponding scores, and calculated the total score. The predicted probability corresponding to the total score was the probability of severe fatigue in patients with CC. The C-Index of the nomogram calculated by Bootstrap method was 0.921 (95% CI: 0.877$\sim$0.958), which indicated that it had good discrimination.

**Fig. 2:**
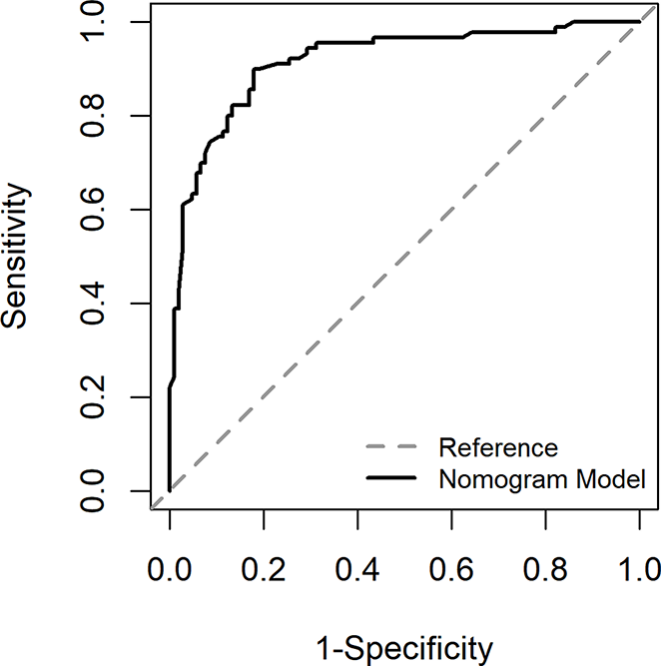
ROC curve of development group

**Fig. 3:**
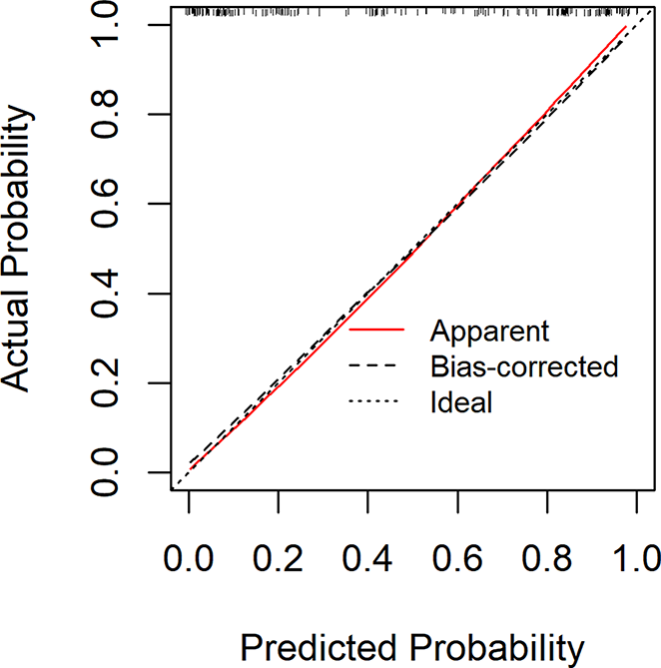
Calibration curve of development group

The ROC curve in Fig. 2 showed that the best cut-off value of the prediction probability of the nomogram was 0.412, which corresponded to the maximum Jordan index of 0.721. At this time, the sensitivity of the model was 0.821, the specificity was 0.900, and the accuracy was 0.857. AUC was 0.916 (95% CI: 0.876$\sim$0.957), which further indicated that the nomogram had high discrimination. The calibration curve in Fig. 3 showed that the predicted probability of the nomogram fitted well with the actual probability. The Hosmer Lemeshow verification showed *χ*^*2*^ = 9.021, *P* = 0.340 > 0.05, further indicating the good calibration of the nomogram.

**Fig. 4:**
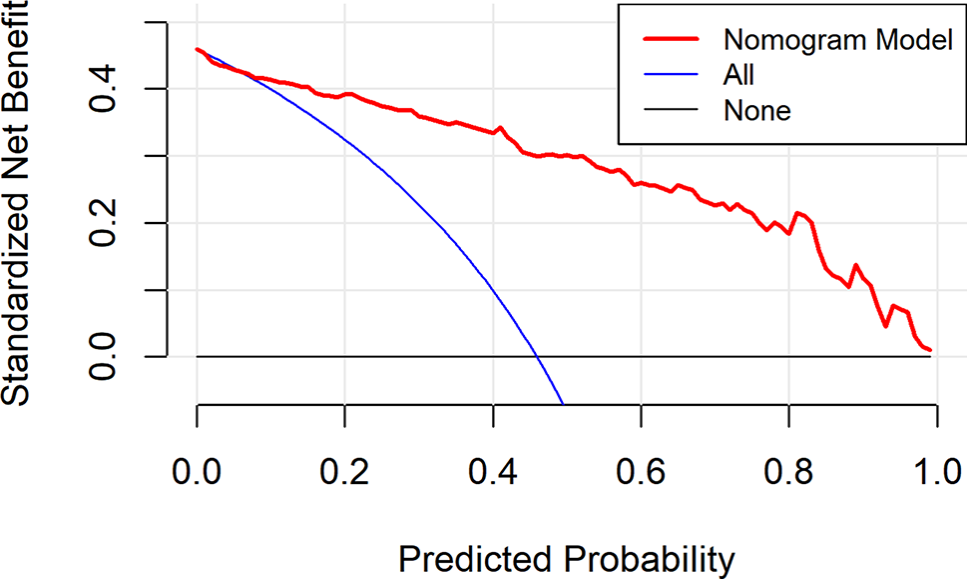
DCA curve of development group

From the DCA curve in Fig. 4, it can be seen that when the prediction probability was greater than about 10%, the benefit from using the nomogram was positive, and there was a wide threshold range, which indicated that the use of the nomogram can benefit better.

### Model validation

**Fig. 5:**
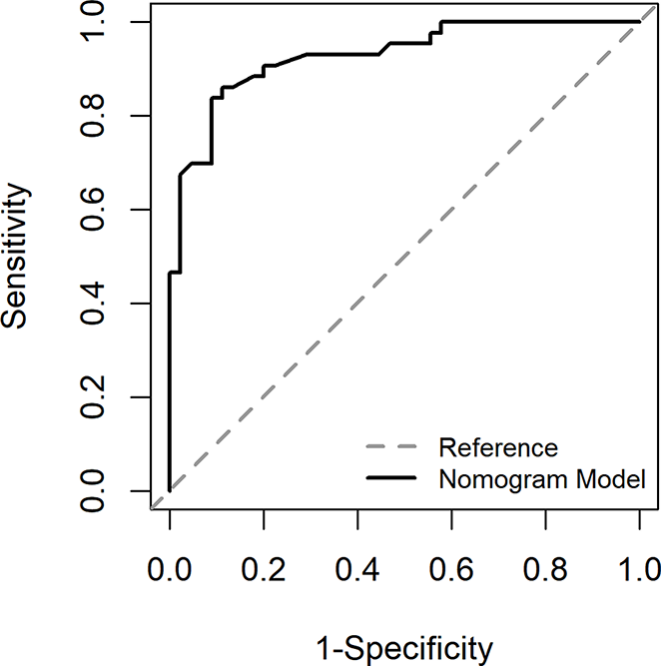
ROC curve of validation group

**Fig. 6:**
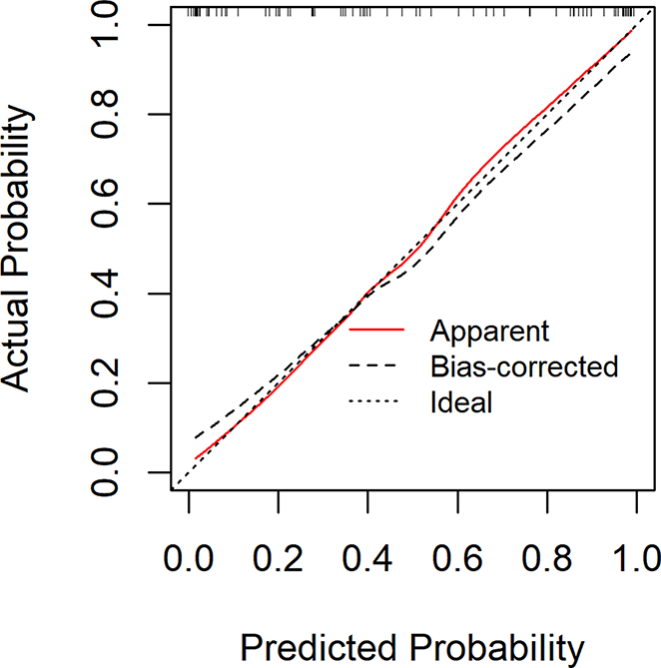
Calibration curve of validation group

**Fig. 7:**
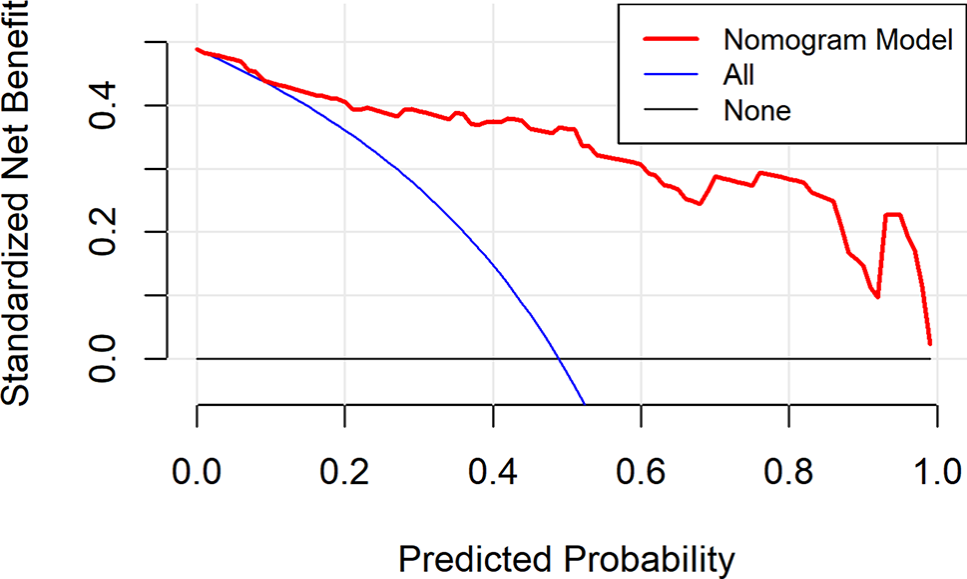
DCA curve of validation group

The nomogram above can be used to calculate the probability of severe CRF of each patient with CC in the validation group, and then the ROC curve (Fig. 5), calibration curve (Fig. 6) and DCA curve (Fig. 7) can be generated according to the probability.

The Fig. 5 showed that the AUC of the area under the ROC curve was 0.928 (0.876$\sim$0.980), and the best cut-off value of the prediction probability of the nomogram model was 0.444, corresponding to the maximum Youden index of 0.748. At this time, the sensitivity of the model was 0.889, the specificity was 0.860, and the accuracy was 0.875, indicating a high degree of differentiation of the nomogram. The Fig. 6 showed that the Calibration calibration curve had good consistency (*χ*^*2*^ = 8.89, *P* = 0.340 > 0.05).

From the DCA curve in Fig. 7, it can be seen that when the prediction probability was greater than about 12%, the benefit of using the nomogram was positive, and it had a wide threshold range, indicating the good clinical practicability of the nomogram.

## Discussion

This study showed 283 patients with CC had CRF of different degrees, and the incidence was as high as 99%, of which the incidence of mild and moderate CRF was 53.2%, and the incidence of severe CRF was 46.8%. Through the nomogram above, we learned that in addition to demographic and clinical characteristics, patients’ psychological conditions were more influential, similar to the model of CRF in patients with breast cancer [17].

### Risk factors of sever CRF in patients with CC

Our study found that long-term passive smoking was risk factor of sever CRF. The reason may be that many carcinogenic and toxic chemicals in second-hand smoke have high concentrations, leading to malignant diseases [46], or passive smoking patients have more negative emotions and poorer sleep disorders, which may exacerbate CRF [47]. We found that tumor recurrence was risk factor of sever CRF. It may be due to patients with tumor recurrence feeling fearful of the disease, suspecting the possibility of curing the disease, affecting their confidence in treatment, and having poor mental health, which in turn exacerbates CRF [48]. Our study also found that negative coping style (avoid or yield) was risk factors for sever CRF. Perhaps it is because negative coping style can affect the recovery process of cancer patients, leading to a cold and negative attitude towards their own diseases. Over time, this can increase the psychological burden on patients and lead to CRF [49].

### Protective factors of sever CRF in patients with CC

Our study found that patients with monthly income > 5000 CNY had a lower risk of severe CRF. Perhaps it is because patients usually face high medical costs after diagnosis, which brings greater psychological pressure to low-income patients [17], and may lead high-risk CRF. We found that patients who exercised ≥ 2–3 times a week had a lower risk of severe CRF. This is because exercise can increase the body’s blood oxygen content, accelerate metabolism, stimulate the central nervous system, and improve the patients’ mental state, thereby eliminating CRF [50]. Our study found that patients who experienced higher social support have relatively lower CRF, which may be due to the social support provided by role relationships helping to stabilize and develop positive self-esteem and confidence, enhancing patients’ ability to withstand stress, and reducing CRF [51]. We also found that patients with higher SOC had a lower risk of developing severe CRF. This is because there are physiological and psychological stressors in the diagnosis and treatment of cancer, and SOC can strengthen the management of corresponding stressors, enabling patients to maintain good physical and mental health outcomes [52].

### Evaluation and analysis of the nomogram

The areas under the ROC curve of both groups were greater than 0.8, indicating that the nomogram can better distinguish severe CRF patients [45]. In the consistency test, the calibration curves were well fitted (*P* < 0.05) in both groups, indicating that the probability of severe CRF predicted by the nomogram was consistent with the actual probability of severe CRF in patients, and the accuracy of prediction was high. The DCA analysis showed that the net benefit of applying the nomogram to most thresholds in both groups was good. According to the best cut-off value 0.444 in ROC curve, patients with CC can be divided into high-risk group and low-risk group of CRF. In addition, this study visualized the the regression equation results in the form of the nomogram, which was more intuitive and convenient for calculation, and was conducive to the practical application of the model in clinical practice [53]. For patients whose prediction probability was close to or higher than the optimal threshold, early intervention could be carried out according to their coping style, social support, SOC and so on.

### Clinical implications

Our study has developed the first nomogram of CRF for patients with CC. It can strengthen the risk identification of severe CRF, and its independent risk factors provided scientific basis for patients to implement intervention measures. For example, if a patient exercises ≥ 2–3 times/week, has a per capita income of > 5000CNY, and has high social support characteristics, their scores for exercise, income, and social support can be calculated based on the nomogram. Then, the above scores are added up to obtain the total score of the patient. Based on the nomogram, estimate the probability of sever CRF occurrence corresponding to the total score, that is, the probability of patient experiencing sever CRF. This nomogram was significant for strengthening risk management, reducing or controlling the occurrence of severe CRF.

### Limitations

The nomogram developed in this study may have the following limitations. Firstly, the predicted results of the nomogram remain unchanged over time, but in fact, the outcomes of disease are changing with improvements in treatment, early detection, and changes in natural history, therefore, over time, the performance of the nomogram may become less accurate. Secondly, although studies have shown that nomogram is superior to the judgment of clinical doctors, however, the conclusion is purely based on AUC and does not equate to improving clinical efficacy. Again, although nomogram can be used to define the effectiveness of clinical trials, treatment decisions for these cases should follow the inclusion criteria determined by the nomogram and the subsequent benefits related to treatment, rather than just the estimated risks in the nomogram. Finally, although the nomogram performs well, the evaluation of whether it can improve patient and doctor satisfaction, and tumor prognosis is often overlooked.

## Conclusion

The nomogram for severe CRF in patients with CC had good identifiability, accuracy and clinical practicality, and can be used as a prediction and evaluation tool for severe cases of clinical patients with CC.

## Data Availability

The datasets generated and/or analyzed during the current study are not publicly available due to the protection of individual privacy of participants. However, these and the methodological tools used may be made available from the corresponding author on reasonable request.

## Abbreviations

CC: Cervical cancer
CRF: Cancer-related fatigue
SOC: Sense of coherence
AUC: Area under curve
DCA: Decision curve analysis
C-Index: Concordance Index
ROC: Receiver Operating Characteristic
